# Effect of Ultraviolet-A (UV-A) and Ultraviolet-C (UV-C) Light on Mechanical Properties of Oyster Mushrooms during Growth

**DOI:** 10.1155/2014/687028

**Published:** 2014-12-14

**Authors:** Tindibale L. Edward, M. S. K. Kirui, Josiah O. Omolo, Richard G. Ngumbu, Peter M. Odhiambo

**Affiliations:** ^1^Department of Physics, Egerton University, Egerton 20115, Kenya; ^2^Department of Chemistry, Egerton University, Egerton 20115, Kenya

## Abstract

This study investigated the effects of ultraviolet-A (UV-A) and ultraviolet-C (UV-C) light on the mechanical properties in oyster mushrooms during the growth. Experiments were carried out with irradiation of the mushrooms with UV-A (365 nm) and UV-C (254 nm) light during growth. The exposure time ranged from 10 minutes to 60 minutes at intervals of 10 minutes and irradiation was done for three days. The samples for experimental studies were cut into cylindrical shapes of diameter 12.50 mm and thickness 3.00 mm. The storage modulus, loss modulus, and loss factor of the irradiated samples and control samples were determined for both UV bands and there was a significant difference between the storage modulus, loss modulus, and loss factor of the irradiated samples by both UV bands with reference to the control sample, *P* < 0.05. UV-C light irradiated samples had higher loss modulus and loss factor but low storage modulus as temperature increased from 35 to 100°C with respect to the control sample while UV-A light irradiated samples had lower loss modulus, low loss factor, and higher storage modulus than UV-C irradiated samples.

## 1. Introduction

The sun emits ultraviolet radiation in the form of ultraviolet-A (UV-A), ultraviolet-B (UV-B), and ultraviolet-C (UV-C) bands [1]. UV-C light is blocked by the ozone layer; hence, of the three UV bands, UV-A and UV-B are the only bands that reach the earth's surface after atmospheric filtration. However, mushrooms grow in dark locations that receive inadequate sunlight, thus creating the need to expose them to ultraviolet light during growth. Mushroom consumption has increased remarkably because of their desirable aroma and taste and high nutritional content [2]. Color, fresh, and clean appearance and uniform closed buttons also have high importance for mushroom quality and consumer preferences [3]. The mechanical properties of the mushrooms produced after irradiation during growth are a very important factor to consider meeting consumers' needs. These properties mainly result from the structure, physical state, and rheology. They are needed for process design, estimating other properties, characterizing foods, and quality determination. Texture is one of the important factors to evaluate quality of mushroom. Undesirably, the stability of texture can be only maintained for a very short period of storage; it is usually changed quickly after harvest [4]. Stiffness, toughness, brittleness, and pliability are considerable characteristics during the analysis of fruit body texture.

Improving mushroom quality and texture as well has been preceded by several methods. The texture [5] was assessed through measuring the force required to shred a bulk sample. A research in which changes through dry matter content were indirectly measured [6] indicated that the texture may be changed due to the changes of cellular materials and moisture loss. A method to measure tissue compressive stiffness was developed where changes in button mushroom texture in different sizes and stages were evaluated [7]. Another method to analyze the texture properties [8] focused on the changes of tenderness, pliability, toughness and brittleness of post harvested and cooked mushroom was established. Previous research revealed some relations between textural characteristics and constituents of fruit bodies like hyphae density which was proved to be one of criteria that determined the stiffness in* A. bisporus* [7]. Another determined stiffness criterion was cell turgidity which was reflected by water content in mushroom [9].

Dynamic mechanical analysis (DMA 983) can be used to gain insight into the factors affecting food quality through simulation of processing conditions. There have been, however, relatively few studies on the dynamic mechanical properties of food, although [10] did perform a comparative DMA study on starches of wheat. In this study, DMA experiments were performed on two food products, commercial white bread and dried pasta. The DMA storage and loss moduli obtained provided valuable information about the softness and keeping properties of bread, as well as the cooking characteristics of pasta.

A research on the influence of temperature of freeze drying process on the mechanical properties of dried mushroom [11] indicated that high value of stress in the fresh mushrooms undergoes high reduction in effect of freeze drying process and the freeze dried material preserves approximately constant stress value in a wide strain range.

A study on anisotropy of mechanical properties of mushroom (*A. bisporus)* was carried out by [12]. Strenght tests, hysteresis tests, and creep tests of mushrooms compressed between two parallel plates were carried out; however, the mushroom sample used had not been exposed to UV-A and UV-C during growth. However, only limited information is reported in the literature about mechanical properties of mushrooms especially oyster (*Pleurotus ostreatus species*) that have been exposed to UV-C and UV-A light during growth.

Viscoelastic materials simultaneously exhibit a combination of elastic and viscous behavior. While all substances are viscoelastic to some degree, this behavior is especially prominent in polymers. There is however limited information in the literature concerning the viscoelastic behavior of mushrooms that have been UV irradiated especially during growth.

Various techniques have been used to study mechanical properties of food. They include Instron 5566 stress testing machine and the dynamic mechanical analysis using the Dynamic Mechanical Analyzer, (DMA 983). Historically, the methods used to evaluate and predict these properties have been somewhat arbitrary and nonquantitative. The use of analytical instrument techniques such as thermal analysis provides a more quantitative, reproducible way for characterizing food products. Dynamic mechanical analysis (DMA), for example, can provide information about the mechanical properties of food and how they are affected by various processing conditions [10].

## 2. Materials and Methods

### 2.1. Growing of Oyster Mushrooms

Wheat grains were prepared for grain spawn by being boiled, drained, filled in containers, and sterilized. The substrate was then prepared from wheat straw and was pasteurized by hot water immersion to kill contaminants. The pasteurized substrate was then spawned after ensuring that the substrate has cooled down to 30°C. The spawn was mixed with the substrate when filling the perforated bags. The bags were thirteen and were labeled B1–B13. Spawn run followed where the mycelium was grown through the substrate. The bags once spawned were placed in a cage that had been prepared where mycelium colonized the substrate in two to three weeks and started to form small fruiting bodies. Near darkness, controlled temperature, and humidity conditions were provided. Humidity was maintained between 80 and 100% by spraying water several times per day and the temperature was maintained between 15 and 25°C.

### 2.2. Exposure of Mushrooms to UV Light during Growth

UV-A light (365 nm) and UV-C light (254 nm) irradiation began once the mushrooms cap started opening from the stem. An 8 W Ultraviolet fluorescent lamp made by UVITEC (model LF-204.LS) was used. The lamp irradiates at the ranges (200–300) nm and (300–400) nm with a switch that shifts between the two ranges. The UV-A intensity was 3.5 W/m^2^ at 365 nm while the UV-C applied intensity was 0.0327 W/m^2^ at 254 nm. The rates of irradiation doses were 0.21 kJ/m^2^/min for UV-A radiation and 1.96 J/m^2^/min for UV-C radiation. B1 was not exposed to UV-A and UV-C light and hence was treated as control experiment. Beginning with the lowest exposure time of 10 minutes for bags B2 and B8 and subsequent 10 minutes increment for the next bag up to 60 minutes for the highest exposed bags (B7 and B13) was done, for UV-C and UV-A, respectively. The UV-A and UV-C light exposure took place for three days. All the irradiations in this experiment were performed under the optimized conditions of temperature 20°C and moisture 80%. The first two growth batches, due to reproducibility, were subjected to same procedure. Once the caps were fully opened and separated from the stem, the mushrooms were ready for harvesting. Harvesting was done by holding the mushrooms by their stalks and breaking them off carefully from the substrate. Samples were picked from each bag and dried.

### 2.3. Dynamic Mechanical Analysis

The samples were then tested for their storage and loss factor using DMA-2980 instrument. DMA 2980 analytical instrument is used to test the physical properties of material. The samples for experimental studies were cut into cylindrical shapes of diameter 12.50 mm and thickness 3.00 mm. Each of the samples was placed on the compressional clamp at a time and subjected to changes in stress induced by an oscillating force. The amplitude and the phase of the displacement in the sample in response to applied oscillating force over a range of temperature were measured. Measurement of loss and storage moduli and the loss factor was obtained directly from the DMA.

The storage modulus gives the amount of energy that the sample stores; the loss modulus gives the amount of energy dissipated by the sample when a sinusoidal force is applied. The loss factor (damping factor) is measured as an angle to indicate the lag between the stress and strain giving information about the samples elastic nature. The storage moduli, loss moduli and loss factors of the samples that had been exposed to UV-A and UV-C light during growth and those had not been exposed (control samples) were tabulated for analysis.

### 2.4. Statistical Analysis

The results were statistically analyzed by analysis of variance (ANOVA, Vassar stats statistical computations). The evaluation of equality of means was carried out by the one-way analysis of variance using the F distribution to assess significance. The data were expressed as means ± SD (standard deviation). The test results were considered significant only after reaching *P* < 0.05. The storage modulus, loss modulus, and loss factor of irradiated sample were compared with the control sample and multiple comparisons were performed using the Turkey test.

## 3. Results and Discussions 

There was a significant difference in storage modulus between the irradiated and control samples, *P* < 0.05. The temperature range was 25–100°C but 35–100°C was chosen for analysis. There was a drop in storage modulus after irradiation of the samples by both UV-A and UV-C light during growth. The samples irradiated by UV-C had a higher average storage modulus drop compared to those treated by UV-A light at different irradiation times from 35 to 100°C as indicated in Table 1.

This indicates that irradiation of samples with both UV light bands lowers storage modulus of the mushrooms. The samples under UV-C and UV-A exhibited similar viscoelastic behavior. The regions included the glassy region in which the samples were hard, springy, or rock-like. In this region, the bending of the bonds was occurring and temperature range was 35–85°C. Glass transition region then followed in which the samples softened and thus became less hard as storage modulus decreased and tan *δ* peaks, temperature ranging from 80 to 95°C.

The samples then started undergoing slippage of main chain (rubbery plateau region) and the temperature ranged from 95 to 100°C. Samples that were irradiated for longer durations (40, 50, and 60 minutes) registered lower values of storage modulus especially for UV-C irradiated samples compared to those that were irradiated for short time intervals (10, 20, and 30 minutes).

The low storage modulus for both UV-C and UV-A irradiated samples as temperature increases (Tables 2 and 3, resp.) can be attributed to low levels of ergosterol which was subjected to photolysis and yielded photoirradiation products, the principal ones being vitamin D_2_, tachysterol, and lumisterol when the mushrooms were irradiated by UV-C and UV-A light during growth. Samples under UV-C irradiation had lower storage modulus as temperature increased because they had higher levels of vitamin D_2_ concentrations and this indicated that most of the ergosterol in these samples had undergone photolysis during irradiation [13]. This means that ergosterol presence in the samples increases the storage modulus since it is a component of the mushrooms cell membrane.

Tables 4 and 5 show the loss modulus for UV-A and UV-C irradiated samples, respectively.

Loss modulus of the mushrooms represents the energy lost as heat and is a measure of vibrational energy that has been converted during vibration and that cannot be recovered. There was a significant difference in the loss modulus between the irradiated samples and control samples, *P* < 0.05. UV-C irradiated samples had a higher increase in loss modulus than UV-A irradiated samples.

Loss factor of mushrooms sample is the measure of the energy lost in terms of the recoverable energy and represents mechanical damping or internal friction in viscoelastic system. There was a significant difference in loss factor between the control and irradiated samples, *P* < 0.05. The samples irradiated by UV-A light during growth recorded a decrease in loss factor with respect to the control sample (Table 6) while those irradiated by UV-C light had an increase in loss factor (Table 7). Irradiation of samples for 60 minutes had no further change on the storage modulus, loss modulus, and loss factor of the samples as samples under this time duration recorded similar values as those under 50 minutes of irradiation. The high values of loss factor in UV-C light treated samples indicated that the mushrooms samples had a nonelastic strain component and the low loss factor of UV-A irradiated samples indicated that the samples had an elastic strain component.

## 4. Conclusion

1. Irradiation of mushrooms during growth with UV-A and UV-C light leads to a decrease in storage modulus with increase in temperature with UV-C irradiated samples having a higher decrease than UV-A irradiated samples.
2. Loss modulus and loss factor decrease with respect to control sample for UV-A light irradiation.
3. For UV-C irradiated samples, the loss modulus and loss factor increase with respect to control sample.
4. UV-C light had a greater impact on the mechanical properties of oyster mushrooms compared to UV-A light. These changes in mechanical properties did not affect the quality of the mushroom.

## Figures and Tables

**Table 1:** The average storage modulus (*E*′) and its percentage drop at different exposure times for UV-A and UV-C light from 35 to 100°C.

Samples irradiation time (minutes)	Average *E*′ (MPa)		Percentage (%) drop in *E*′	
	UV-A	UV-C	UV-A	UV-C
Control sample	8.987	8.987	—	—
10	7.614	6.850	15	23
20	6.367	5.760	29	36
30	5.874	5.540	35	38
40	5.506	5.250	39	42
50	5.269	5.046	41	44
60	5.269	5.046	41	44

**Table 2:** Storage modulus (MPa) for control sample (B1) and UV-C irradiated oyster mushroom samples at different temperatures (°C).

Temperature (°C)	Storage modulus (MPa)					
	Sample B1	B2	B3	B4	B5	B6 and B7
35	11.18	8.85	7.35	7.25	6.50	6.37
40	11.00	8.20	7.15	7.00	6.30	6.14
45	10.67	8.10	6.86	6.50	6.25	6.00
50	10.39	7.95	6.75	6.30	6.20	5.90
55	10.32	7.75	6.33	6.05	5.85	5.62
60	10.17	7.50	6.15	5.90	5.75	5.52
65	10.10	7.40	6.05	5.75	5.50	5.40
70	10.06	7.20	5.75	5.60	5.30	5.20
75	10.02	7.10	5.55	5.45	5.30	5.10
80	9.960	6.80	5.40	5.30	5.10	4.80
85	8.540	5.95	5.20	5.10	4.75	4.50
90	5.210	4.50	4.30	4.20	3.80	3.60
95	4.900	4.40	3.95	3.70	3.50	3.30
100	4.900	4.20	3.90	3.50	3.41	3.20

**Table 3:** Storage modulus (MPa) for control sample (B1) and UV-A irradiated oyster mushroom samples at different temperatures (°C).

Temperature (°C)	Storage modulus (MPa)					
	Sample B1	B8	B9	B10	B11	B12 and B13
35	11.18	9.87	7.77	7.17	6.70	6.63
40	11.00	9.69	7.57	7.12	6.62	6.51
45	10.67	9.56	7.50	6.91	6.46	6.34
50	10.39	9.28	7.31	6.80	6.42	6.31
55	10.32	9.11	7.20	6.70	6.40	6.16
60	10.17	8.87	7.19	6.60	6.20	6.03
65	10.10	8.45	7.15	6.50	6.11	5.86
70	10.06	8.33	7.13	6.33	6.03	5.54
75	10.02	8.15	7.04	6.30	5.72	5.35
80	9.960	7.92	6.95	6.30	5.55	5.21
85	8.540	6.13	6.05	5.65	5.44	4.89
90	5.210	4.88	4.27	4.15	4.03	3.86
95	4.900	3.20	3.03	2.88	2.71	2.55
100	4.900	3.19	2.98	2.86	2.70	2.53

**Table 4:** Loss modulus (MPa) for control sample (B1) and UV-A irradiated oyster mushroom samples at different temperatures (°C).

Temperature (°C)	Loss modulus (MPa)					
	Sample B1	B8	B9	B10	B11	B12 and B13
35	1.40	1.20	0.90	0.72	0.60	0.59
40	1.40	1.23	0.90	0.72	0.60	0.59
45	1.40	1.25	0.90	0.72	0.60	0.59
50	1.45	1.28	0.90	0.72	0.60	0.59
55	1.47	1.30	0.90	0.72	0.60	0.59
60	1.50	1.35	0.95	0.72	0.60	0.59
65	1.60	1.45	1.10	0.75	0.60	0.59
70	1.70	1.50	1.25	0.80	0.64	0.59
75	1.85	2.00	1.40	1.10	0.90	0.59
80	2.70	3.40	2.50	1.60	1.15	0.68
85	4.10	3.00	2.60	1.70	1.40	0.53
90	3.60	2.70	2.00	1.50	1.30	0.53
95	2.75	1.65	1.36	1.00	0.78	0.53
100	2.50	1.60	1.25	0.90	0.70	0.53

**Table 5:** Loss modulus (MPa) for control sample (B1) and UV-C irradiated samples against temperature (°C).

Temperature (°C)	Loss modulus (MPa)					
	Sample B1	B2	B3	B4	B5	B6 and B7
35	1.4	1.72	1.59	1.57	1.55	1.54
40	1.4	1.72	1.59	1.57	1.55	1.54
45	1.4	1.72	1.62	1.57	1.55	1.54
50	1.45	1.68	1.65	1.56	1.55	1.54
55	1.47	1.81	1.73	1.61	1.55	1.56
60	1.50	1.92	1.88	1.73	1.68	1.62
65	1.60	1.98	1.94	1.85	1.80	1.75
70	1.70	2.85	2.78	2.70	2.67	2.61
75	1.85	3.50	3.35	2.90	2.92	2.80
80	2.70	3.61	3.41	2.84	2.81	2.71
85	4.10	3.40	3.11	2.79	2.75	2.58
90	3.60	3.12	2.71	2.63	2.59	2.47
95	2.75	2.71	2.59	2.51	2.46	2.40
100	2.50	2.54	2.41	2.37	2.35	2.25

**Table 6:** Loss factor for control sample (B1) and UV-A-irradiated oyster mushroom samples at different temperatures (°C).

Temperature (°C)	Loss factor					
	Sample B1	B8	B9	B10	B11	B12 and B13
35	0.13	0.12	0.12	0.09	0.09	0.08
40	0.13	0.12	0.12	0.09	0.09	0.08
45	0.13	0.13	0.12	0.09	0.09	0.09
50	0.13	0.14	0.12	0.09	0.09	0.09
55	0.14	0.14	0.13	0.11	0.09	0.09
60	0.15	0.15	0.13	0.11	0.09	0.09
65	0.16	0.17	0.15	0.12	0.09	0.10
70	0.17	0.18	0.16	0.12	0.11	0.11
75	0.19	0.25	0.17	0.17	0.16	0.11
80	0.27	0.43	0.35	0.25	0.21	0.13
85	0.48	0.49	0.43	0.30	0.25	0.11
90	0.69	0.55	0.47	0.37	0.32	0.14
95	0.67	0.51	0.45	0.34	0.29	0.20
100	0.60	0.50	0.42	0.32	0.27	0.20

**Table 7:** Loss factor for control sample (B1) and UV-C irradiated oyster mushroom samples at different temperatures (°C).

Temperature (°C)	Loss factor					
	Sample B1	B2	B3	B4	B5	B6 and B7
35	0.13	0.19	0.20	0.22	0.24	0.24
40	0.13	0.20	0.22	0.22	0.25	0.25
45	0.13	0.21	0.23	0.24	0.25	0.26
50	0.13	0.21	0.24	0.25	0.25	0.26
55	0.14	0.23	0.27	0.26	0.26	0.26
60	0.15	0.26	0.30	0.29	0.29	0.29
65	0.16	0.27	0.32	0.32	0.32	0.32
70	0.17	0.39	0.50	0.48	0.50	0.50
75	0.19	0.49	0.6	0.53	0.55	0.55
80	0.27	0.53	0.63	0.54	0.55	0.56
85	0.48	0.57	0.6	0.55	0.58	0.63
90	0.69	0.69	0.63	0.62	0.68	0.68
95	0.67	0.61	0.65	0.68	0.70	0.73
100	0.60	0.60	0.62	0.67	0.69	0.70
